# Exploring second language students’ language assessment literacy: impact on test anxiety and motivation

**DOI:** 10.3389/fpsyg.2024.1289126

**Published:** 2024-02-12

**Authors:** Fanrong Weng, Xingnan Liu

**Affiliations:** ^1^Department of English, Faculty of Arts and Humanities, University of Macau, Taipa, Macao SAR, China; ^2^Independent Researcher, Fuzhou, China

**Keywords:** language assessment literacy, test anxiety, motivation, second language learning, higher education, multilevel analysis, mainland China

## Abstract

**Introduction:**

This research aims to investigate the impact of students’ language assessment literacy (LAL) on their cognition. The study specifically examines how different levels of LAL influence two critical factors: test anxiety and motivation to learn a second language.

**Methods:**

To achieve the research objectives, a questionnaire was administered to a sample of 415 university students in China. The questionnaire utilized a five-point Likert scale to assess students’ levels of LAL, test anxiety, and motivation to learn a second language. Descriptive data were examined to reveal students’ proficiency in LAL, along with their levels of test anxiety and motivation. Multilevel regression analyses were performed using Mplus to investigate whether students’ LAL proficiency can predict their levels of test anxiety and motivation.

**Results:**

The findings indicated that the participating students had a proficiency level of approximately 60% in the content of the LAL questionnaire. The analysis further revealed the relationships between specific dimensions of LAL and both test anxiety and second language motivation. The multilevel regression analysis suggested that theoretical knowledge about language and language learning, the understanding of the impact and social value of language assessments, and the uses of assessments to enhance learning, positively predicted students’ extrinsic motivation. Furthermore, students’ understanding of the uses of assessments to learn and their theoretical knowledge about language learning were identified as positive predictors of intrinsic motivation. Additionally, it was observed that students’ LAL did not significantly predict test anxiety.

**Discussion:**

These findings emphasize the significance of enhancing students’ LAL due to the identified relationships between LAL dimensions and motivation to learn a second language. The study suggests pedagogical implications for improving LAL, with a focus on specific dimensions that positively impact students’ motivation. The absence of a significant relationship between LAL dimensions and test anxiety prompts further exploration and consideration of additional factors influencing students’ anxiety in language assessments.

## 1 Introduction

Language assessment literacy (LAL), commonly referred to as assessment stakeholders’ understanding of the knowledge, beliefs, and principles regarding language assessment, holds significant significance in language teaching and assessment contexts (Crusan et al., 2016). Previous studies have mainly focused on the LAL of teachers and education administrators (Brindley, 2001; Fulcher, 2012; Baker et al., 2014; Baker, 2016). In recent times, scholars have increasingly directed their attention to the LAL of students, a crucial group of stakeholders in the language learning process. This is because in order to enhance the effectiveness of learning, students are encouraged to assess their own work, identify strengths and weaknesses, and determine strategies for further improvement (Sadler, 2009; Smith et al., 2013). An important aspect of this evaluation process involves students assessing whether they have successfully fulfilled the required tasks and if their responses align with the objectives of the assessment (Sadler, 2010). How well students comprehend the assessment objectives and relevant procedures can impact their learning and goal setting (Smith et al., 2013). Therefore, enhancing students’ LAL is an inevitable step to facilitate their foreign language learning. To date, a few studies have been undertaken to investigate students’ LAL, including examining students’ LAL levels and how students perceive language assessment. However, how students’ LAL impacts their language learning, such as their cognitive factors in language learning, has not yet been given attention.

During students’ language learning process, a variety of cognitive factors can exert significant influences. Among these, motivation and test anxiety stand out as considered mutable factors (Cheng et al., 2014). Motivation and test anxiety are significantly correlated with students’ performance in language tests. Previous studies have indicated that the increased motivation and the decreased test anxiety could help students demonstrate their real language competency in language tests (Wu and Lee, 2017; Dörnyei and Ushioda, 2021; Sun and Zhang, 2022). Furthermore, prior studies have identified a significant association between motivation and test anxiety (Cheng et al., 2014; Khalaila, 2015). As a result, in order to facilitate students’ language learning and test performance, it becomes crucial to investigate these two variables concurrently and explore potential approaches for improving students’ motivation without exacerbating test anxiety.

So far, scant attention has been paid to examining whether and how students’ LAL levels can impact their test anxiety and second language (L2) motivation, both of which are closely associated with language learning. Therefore, to fill the existing research gap, the present study intends to explore students’ LAL levels and their potential influences on cognitive factors, specifically test anxiety and motivation. Four hundred and fifteen students from seven universities in China participated in this study, responding to the questionnaire. Descriptive analysis and multilevel regression analyses were conducted to explore the students’ LAL level and their impacts.

## 2 Literature review

### 2.1 Student assessment literacy

Assessment literacy (AL) was first proposed by Stiggins (1991), who defined it as “a basic understanding of the meaning of high-and low-quality assessment” and the ability “to apply that knowledge to various measures of student achievement” (p. 535). Since then, AL has garnered considerable attention from teachers, school administrators, and other stakeholders. Previous research has proved that assessment-literate stakeholders are more capable of making informed decisions regarding assessment practices and score interpretation (Harding and Kremmel, 2016; Xu and Brown, 2016; Weng and Shen, 2022).

Prior research has primarily focused on the AL of teachers and university or school administrators (Fulcher, 2012; Baker et al., 2014; Crusan et al., 2016; Weng and Shen, 2022; Weng, 2023). There has been a shift towards exploring students’ AL, and researchers have attempted to define or conceptualize students’ AL (Price et al., 2012; Smith et al., 2013; Butler et al., 2021). For example, Price et al. (2012) underlined that for students to enhance their assessed performance, they needed to better understand assessment approaches, criteria, and the association between assessment and learning. In another study, Smith et al. (2013) defined student AL as students’ understanding of assessment standards, their utilization of assessment results to support their learning, and their ability to perform according to the assessment criteria in their context. Furthermore, Smith et al. (2013) conceptualized student AL in terms of three aspects. First, students should be aware of the objective of assessment and how it can relate to their own study. Second, they should comprehend the assessment procedures and how these procedures might influence students’ ability to complete the assessment. Lastly, students should be capable of evaluating their assessment performance and the ways to refine it.

Several studies have investigated students’ AL levels (Kremmel and Harding, 2020; Butler et al., 2021; Yan and Fan, 2021). For instance, Butler et al. (2021) examined Chinese primary school students’ perceptions and attitudes towards assessment. It was found that these children were able to elaborate their preferences and attitudes toward language assessment. Yan and Fan (2021) investigated the LAL levels of PhD candidates specializing in language testing and found that they had significantly higher levels of LAL than their peers majoring in language pedagogy. Additionally, Kremmel and Harding (2020) developed and validated an LAL survey to examine the LAL needs of various stakeholder groups, including students. This LAL survey was developed based on Taylor’s (2013) LAL models with nine dimensions, including but not limited to knowledge of theory, language pedagogy, impact and social values, scores and decision-making.

Several studies have focused on interventions which aimed at improve students’ AL (Smith et al., 2013; Deeley and Bovill, 2017; Torshizi and Bahraman, 2019). For example, Deeley and Bovill’s (2017) case study of 33 undergraduate social sciences students in Scotland demonstrated that a staff–student partnership in assessment not only enhanced students’ learning agency but also developed their AL. In addition, Torshizi and Bahraman (2019) conducted a study with 36 English major undergraduate students in Iran. The interview data revealed that learning through teaching can improve students’ AL and deep learning. Additionally, Smith et al. (2013) conducted a pseudo-experimental study and discovered that a 50-min training program effectively increased students’ AL levels and improved their assessment outcomes.

Taking into account the varied characteristics of students’ AL across different fields, it is crucial to investigate it within specific contexts, such as the context of teaching English as an L2, a second language, which is prevalent in non-English speaking countries and involves various language assessments. However, among the aforementioned studies, only four have paid attention to students’ AL in language assessment context, that is, students’ LAL (Torshizi and Bahraman, 2019; Kremmel and Harding, 2020; Butler et al., 2021; Yan and Fan, 2021). Existing research on students’ LAL has primarily focused on their perceptions of language assessment and ways to improve their LAL. However, the impact of students’ LAL on their language learning and related cognitive factors, such as test anxiety and motivation, have received limited attention.

### 2.2 Test anxiety

Test anxiety is one of the key concerns in the education field and has been a research focus of language teaching and learning (e.g., Hill and Wigfield, 1984; Stöber and Pekrun, 2004). It refers to the inclination to respond to assessment with tension, worries, intrusive thoughts, and physiological arousal (Spielberger and Vagg, 1995). Test anxiety is considered a multidimensional construct, encompassing cognitive, affective, behavioral, social, physiological, and motivational dimensions (e.g., Sarason et al., 1964; Spielberger and Vagg, 1995; Tan and Pang, 2023). Recent studies have empirically examined students’ test anxiety and found that test anxiety was negatively associated with students’ test performance and academic achievement (Putwain and Daly, 2013; Khalaila, 2015; Chin et al., 2017; Roick and Ringeisen, 2017).

Several studies have investigated the factors associated with test anxiety using self-reported instruments (e.g., Trifoni and Shahini, 2011; Kurbanoğlu and Nefes, 2015). High-stakes testing is one significant factor when it comes to test anxiety. High-stake testing is an important factor that contributes to test anxiety, because the awareness of the importance of such tests could add to test-takers’ test anxiety levels and impede them from performing to their full capacity (Bertrams et al., 2013; Kavakci et al., 2014; Kurbanoğlu and Nefes, 2015). Insufficient preparation for tests accounts for another factor that increases test anxiety (Trifoni and Shahini, 2011; Saha, 2014; Alemu and Feyssa, 2020). Furthermore, misconceptions regarding test-taking skills, misunderstandings of learning strategies, and poor time management skills could all contribute to test anxiety (Mealey and Host, 1992; Schnitzer, 1998; Beggs et al., 2011).

As language tests have unique features from the tests of other subjects, the language test anxiety should be studied separately. In this study, language test anxiety refers to the anxiety related to language assessment contexts. Several studies have examined the association between test anxiety and language test performance. Several studies have identified a negative association (Rezazadeh and Tavakoli, 2009; Tsai and Li, 2012; Wu and Lee, 2017), while others have found no significant impact of test anxiety on language test performance (In’nami, 2006; Huang, 2018). Furthermore, a few studies have explored interventions to alleviate language test anxiety for language learners (Lee et al., 2015; Tasan et al., 2021). For example, the experimental study by Tasan et al. (2021) with 140 sophomore-year English language learners in Turkish universities found that practicing pranayama breathing could help students mitigate their test anxiety levels. Another experimental study by Lee et al. (2015) discovered that audio-visual aids were effective in reducing L2 learners’ test anxiety in listening tests.

The abovementioned studies have identified the causes of test anxiety and examined the interventions aimed to alleviate the language test anxiety of language learners, involving physical exercises or external aids. However, few studies have examined how language students can independently overcome test anxiety, such as by enhancing their understanding of tests, adopting appropriate test preparation strategies, and altering their learning behaviors.

### 2.3 L2 learners’ motivation

Motivation is considered as a significant factor in L2 acquisition and has an essential impact on students’ development of L2 abilities (Dörnyei and Ushioda, 2021). So far, the development of motivation has been studied from various perspectives, such as attribution theory, expectancy-value theory, and self-determination theory (Deci and Ryan, 1985a; Weiner, 1986; Wigfield and Eccles, 2000; Ryan and Deci, 2000a). Among these different theories, the self-determination theory proposed by Ryan and Deci (2000a) can serve as a useful theoretical framework for examining the motivation of L2 learners in this study.

The self-determination theory classifies motivation along a continuum of self-determination, including three major types: amotivation, extrinsic motivation, and intrinsic motivation. Amotivation refers to the absence of an incentive to act. Extrinsic motivation refers to engaging in an activity driven by rewards or other external factors, rather than the enjoyment of the activity itself (Ryan and Deci, 2000c). According to varying degrees of autonomy, extrinsic motivation can be further divided into external regulation (the least autonomous), introjected regulation, identified regulation, and integrated regulation (the most autonomous). Intrinsic motivation, on the contrary, is defined as “the inherent tendency to seek out novelty and challenges, to extend and exercise one’s capacities, to explore, and to learn” (Ryan and Deci, 2000b, p. 70). In educational settings, it is desirable for students to have sufficient intrinsic motivation, because it will allow them to naturally enjoy learning and accomplishing long-term goals (Ryan and Deci, 2000b,c). However, this ideal situation will not remain sustained for a long time, because students’ intrinsic motivation diminishes when they are required to fulfill extrinsic requirements during the learning process (Dörnyei and Ushioda, 2021). Therefore, supportive strategies are necessary to preserve and enhance students’ intrinsic motivation.

L2 motivation plays a vital role in L2 learning, and it is challenging for students with insufficient motivation to achieve their long-term goals (Dörnyei, 1998). Numerous studies have identified the factors that influence students’ L2 motivation, such as exams (Sugita, 2007; Nakata, 2010), the delivery methods of course material (Henry, 2019; Chen, 2020; Chen and Kent, 2020), learning environment and pedagogy (Yu and Geng, 2020; Weng et al., 2023), and teachers’ interpersonal variables and behaviors (Yélamos-Guerra et al., 2022). The abovementioned factors are relevant to teachers’ instructional approaches. However, the impact of students’ understanding of instruction and assessment, such as students’ LAL, on students’ L2 motivation, has not yet been investigated.

## 3 Aim of the study

According to a recent literature review, it is evident that a research gap exists in the current understanding of students’ LAL. Current studies have primarily concentrated on conceptualizing students’ LAL and identifying the factors that contribute to LAL, while few studies have explored the potential influence of students’ LAL on their test anxiety and motivation related to their language learning. To fill this gap, the present study intends to examine students’ levels of LAL, as well as its potential influences on their test anxiety and motivation, two mutable and correlated factors that may collectively influence the language learning process. This study is guided by the following research questions:

*RQ 1*:What are the overall self-reported levels of LAL among university students, and how do these levels vary across the four dimensions of LAL?

*RQ 2*:What are the levels of self-reported test anxiety and motivation among university students?

*RQ 3*:To what degree do the LAL dimensions of students predict test anxiety?

*- Hypothesis 1: LAL dimensions will negatively and significantly predict students’ test anxiety*.

*RQ 4*:To what degree do students’ LAL dimensions predict L2 motivation?

*- Hypothesis 2: LAL dimensions will positively and significantly predict students’ L2 motivation (i.e., external regulation, identified regulation, introjected regulation, knowledge, acknowledgement, stimulation).*

## 4 Method

### 4.1 Context and participants

This research was conducted in China, where the exam-oriented culture is widespread, and English language education holds significant importance in the Chinese education system. Nearly all Chinese university students are obligated to study English for 6 to 9 years during their K-12 stage and must undergo the English College Entrance Examination (gaokao) for university admission. At the university level, English majors are required to take Tests for English Majors (level 4 and level 8), while non-English major students are mandated to enroll in English courses during their initial 2 years of college to pass various English tests (such as the National College English tests) for graduation and to pursue future career or academic opportunities.

Purposeful sampling was used to collect responses from Chinese university students. To recruit students from diverse university tiers, online questionnaires were distributed among seven universities representing different tiers in China, including one top-tier universities (key public universities), three mid-tier public universities (other public universities), and three third-tier universities (private universities). In total, 415 university students, who aged from 18 to 21, responded to the questionnaire. The participants consisted of 290 females and 125 males, with 48 students coming from top-tier public universities, 240 from second-tier public universities, and 127 from private universities. Among the participants, 82 students were English major students, while the remaining 333 students were pursuing other fields of study.

### 4.2 Instruments

This study incorporated a questionnaire with four sections. The first three sections measured students’ LAL levels (20 items), motivation (21 items), and test anxiety (27 items). A five-point Likert scale, ranging from 1 (strongly disagree) to 5 (strongly agree), was employed to gather students’ responses. The fourth section aimed to investigate students’ demographic information, such as their ages and genders.

#### 4.2.1 Students’ LAL

Kremmel and Harding’s (2020) validated LAL survey was employed in this study to assess students’ LAL levels. The internal consistency of the survey dimensions, as indicated by Cronbach’s alpha values, exceeded 0.9. As mentioned in the literature review section, Kremmel and Harding’s (2020) survey was developed based on Taylor’s (2013) LAL model, which includes nine dimensions of LAL relevant to various language assessment stakeholders. According to the characteristics of Chinese university students, four dimensions with 20 items from Kremmel and Harding’s survey were adopted and adapted. The four dimensions focused on (i) theoretical knowledge about language and language learning (5 items), (ii) the uses of assessments to enhance learning (6 items), (iii) the impact and social values of language assessment (4 items), and (iv) the understanding of assessment scores and decision-making (5 items).

The reason for adapting Kremmel and Harding’s LAL survey in this study is that this survey has been tried-and-tested and is applicable in a wide-range of stakeholder groups. The following is one sample item: I am knowledgeable about how to interpret what a particular score says about an individual’s language ability (the understanding of scores and decision-making).

#### 4.2.2 L2 motivation

Noels et al’s. (2000) validated Language Learning Orientations Scale was adapted according to Chinese university contexts in order to measure students’ L2 motivation in this study. The development of this scale was informed by the self-determination theory of Deci and Ryan (1985a). This adapted scale consists of 7 subscales and 21 items, with each subscale consisting of 3 items. One subscale focuses on students’ amotivation. Three subscales measure students’ extrinsic L2 motivation, which includes external regulation, introjected regulation, and identified regulation. External regulation refers to actions that are determined by factors outside of an individual, such as tangible rewards or costs. Introjected regulation involves motivations for engaging in an activity driven by internalized pressures within oneself, leading individuals to compel themselves to undertake that particular activity to avoid guilt or anxiety. Identified regulation is the most self-determined type of extrinsic motivation. In this context, individuals expend effort on an activity because they have personally chosen to do so for reasons that are relevant to them. In the case of students, this could involve engaging in an activity because they recognize its significance in achieving a valued goal (Noels et al., 2000).

In addition, three subscales assess students’ intrinsic motivation, including knowledge, accomplishment, and stimulation. Knowledge is defined as the motivation to engage in an activity driven by the desire to experience the feelings associated with exploring new ideas and acquiring knowledge. Accomplishment refers to the feelings associated with striving to excel in a task or reach a particular goal. Stimulation involves motivation that arises solely from the sensations evoked by engaging in a task, such as experiencing aesthetic appreciation, fun, and excitement (Noels et al., 2000). The following is one sample item: I study an L2 in order to have a better salary later on (external regulation).

The original Language Learning Orientations Scale adopted a 7-point Likert scale. In the current study, however, it has been modified to a 5-point Likert scale. While the 7-point Likert scale can potentially provide a more precise measurement of respondents’ motivation levels, the adaptation to 5-point Likert scale is intended to alleviate the cognitive load on respondents, ultimately enhancing their completion rates. Moreover, according to previous studies, the results of Bendig (1954) reveal no significant differences in the reliability of rating scales spanning three to nine categories. Similarly, the findings from Komorita and Graham (1965) suggest that the reliability of a scale remains unaffected by the number of item scale points. Given that the categories of rating scales appear to have limited impact on the scale reliability, the researchers adopted a 5-point Likert scale for the Language Learning Orientations Scale in this study.

#### 4.2.3 Test anxiety

The Cognitive Test Anxiety Scale, comprising 27 items, was revised and employed in this study to investigate the cognitive dimension of students’ test anxiety (Cassady and Johnson, 2002). This scale was developed based on various validated test anxiety scales, including the inventories of Sarason (1984), Spielberger and Vagg (1995), and Benson et al. (1992), in order to measure the power of a single-factor model of cognitive test anxiety. It assesses students’ reactions to their tests, including their bodily symptoms, tensions, test-irrelevant thinking, and test procrastination. In this study, the wording and content of the items were revised to assess students’ anxiety specifically in the context of language tests. For example, one item was rephrased as follows: “During language tests, I find myself thinking of the consequences of failing.” Furthermore, the original Cognitive Test Anxiety Scale, which initially adopted the 4-point Likert scale, was modified in this study to use a 5-point Likert scale. The adjustment was made to align with the scales used for assessing the students’ LAL scale and L2 motivation in this study. The rationale behind this modification was to maintain a consistent 5-point format across all scales, aiming to streamline the questionnaire, reduce the cognitive load on respondents, improve completion rates, and ensure the consistency in subsequent analyses.

#### 4.2.4 The reliability and construct validity of the instruments

In the present study, the aforementioned scales showed adequate reliability and construct validity. The Cronbach’s alphas for the three scales were 0.972 (LAL scale), 0.942 (test anxiety), 0.901 (motivation); and the Cronbach’s alphas for the different subscales were all higher than 0.88. These results indicated high reliability of these scales. In addition, Table 1 presents the results of the confirmatory factor analyses (CFAs), which further confirmed the scales’ psychometric properties. Regarding the LAL scale, the CFI stands at 0.946, and the TLI at 0.938, indicating an adequate model fit (Marsh et al., 2012). Although the RMSEA value is 0.08, suggesting a fit somewhat less than ideal, it still remains below 1, which is considered acceptable (Kline, 1994). Regarding the motivation questionnaire, the CFI is 0.964, the TLA is 0.954, and RMSEA is 0.069, also indicating an adequate model fit (Marsh et al., 2012).

**Table 1:** Fit statistics for the students’ LAL scale and motivation scale.

	CMIN/*df*	TLI	CFI	RMSEA	SRMR
LAL	3.675	0.938	0.946	0.080	0.037
Motivation	3.002	0.954	0.964	0.069	0.033
Test anxiety	12.191	0.570	0.603	0.164	0.156

### 4.3 Procedures

In April 2022, the researcher contacted the teachers who were teaching English in the seven target universities and requested them to facilitate administering the online questionnaire to their students during class breaks. Before filling out the questionnaire, students were informed of the purpose of this research, their right to voluntarily participate, the option to withdraw from the study at any point, and the confidentiality of the data. Students were assured that their participation would have no impact on their course grades.

### 4.4 Data analysis

The collected questionnaires were analyzed using SPSS 23.0, AMOS 27, and Mplus 8. First, as mentioned above, Cronbach’s alpha tests and CFAs were performed through SPSS 23.0 and AMOS 27 to assess the reliability and the construct validity of the scale. Normality tests were also performed to ensure the normal distributions of the data. Second, descriptive analyses were conducted through SPSS 23.0 to measure students’ LAL, motivation, and test anxiety levels.

Third, following Marsh et al.’s (2012) recommendation, we utilized Mplus 8 to conduct multilevel CFA for seven structural models examined in the study to test the Hypothesis 1 and Hypothesis 2. We applied conventional cutoff criteria that indicate excellent and adequate fit to the data, as recommended by Byrne (2011): (1) CFI (comparative fit index) and TLI (Tucker–Lewis index) ⩾ 0.95 and ⩾ 0.90; (2) RMSEA (root mean square error of approximation) ⩽ 0.06 and ⩽ 0.08.

Finally, Mplus 8 was utilized to perform multilevel regression analysis to explore the possible predictive association between students’ LAL dimensions and both test anxiety and motivation. This examination sought to understand the extent to which LAL dimensions could serve as predictors for these factors. Given that participants in this study are students nested within diverse universities, a “complex” analysis type was adopted. Multilevel modeling approach with Mplus 8 was employed to test our hypotheses, as it is particularly apt for analyzing data structures that are hierarchically nested. Students’ institution was included as a potentially confounding covariate because students from different levels of university tend to report different understandings of LAL, test anxiety, and motivation.

## 5 Results

### 5.1 Descriptive analyses

To answer the first and the second research question, the descriptive results were calculated on the subscale tests and reported in Table 2. The average score for LAL was calculated as 66.37 out of a maximum score of 100. To be more specific, the participants demonstrated similar proficiency levels across four aspects: using assessments to enhance learning (mean score = 3.380), interpreting scores and making decisions (mean score = 3.302), mastering theoretical knowledge about language and language learning (mean score = 3.284), and understanding the impact and social values of language assessment (mean score = 3.288).

**Table 2:** Students’ LAL levels.

	Variables	Mean	*SD*	*α*
1	Uses of assessments to enhance learning	3.380	0.753	0.927
2	Scores and decision making	3.301	0.763	0.915
3	Theoretical knowledge	3.284	0.818	0.923
4	Impact and social values	3.289	0.813	0.930
5	Total LAL	66.370	14.51	0.972

In addition, descriptive analyses were conducted to measure students’ test anxiety and motivation levels, and the results were presented in Table 3. Students’ average test anxiety level is 78.55 out of a maximum score of 135. Furthermore, these students displayed a higher level of average intrinsic motivation than extrinsic motivation.

**Table 3:** Students’ test anxiety and motivation.

Variables	Mean	*SD*	*α*
Test anxiety	78.550	17.886	0.942
Amotivation	2.267	1.070	0.941
External regulation	3.271	0.987	0.890
Introjected regulation	2.841	1.080	0.885
Identified regulation	3.712	0.834	0.880
Intrinsic motivation-knowledge	3.512	0.896	0.915
Intrinsic motivation-accomplishment	3.672	0.886	0.935
Intrinsic motivation-stimulation	3.295	1.000	0.933

### 5.2 Correlation and regression analyses

#### 5.2.1 Correlation analysis

To answer research question three and four, the required assumptions analyses were conducted first to examine the linear relationships between the dependent variable and each independent variable, as well as to assess homoscedasticity, non-multicollinearity, the absence of significant outliers, and the normal distribution of data. No significant violations were found in these analyses. After the initial analyses, Pearson correlation analyses were conducted to explore the relationships between LAL dimensions and other variables, namely test anxiety and motivation.

Table 4 shows that students’ LAL dimensions were significantly and positively associated with their test anxiety levels, with the correlations ranging from 0.322 to 0.444, *p* ≤ 0.01. Moreover, significant positive correlations also existed between almost all LAL dimensions and L2 motivation constructs. Specifically, stronger correlations were found between the LAL dimensions and intrinsic motivation (0.384 ≤ *r* ≤ 0.520, *p* ≤ 0.01), while correlations with extrinsic motivation were ranging from 0.173 to 0.391 (*p* ≤ 0.01).

**Table 4:** Correlations among LAL, test anxiety, and motivation.

Inventory and Scales		LAL survey				Test anxiety	L2 motivation						
							*Amotivation*	*Extrinsic motivation*			*Intrinsic motivation*		
		1	2	3	4	5	6	7	8	9	10	11	12
*LAL survey*													
1	Uses of assessments to learn	–	0.831^**^	0.748^**^	0.743^**^	0.322^**^	0.047	0.173^**^	0.206^**^	0.391^**^	0.477^**^	0.412^**^	0.488^**^
2	Scores and decision-making		–	0.857^**^	0.843^**^	0.429^**^	0.146^**^	0.196^**^	0.253^**^	0.350^**^	0.487^**^	0.384^**^	0.487^**^
3	Theoretical knowledge			-	0.846^**^	0.444^**^	0.143^**^	0.271^**^	0.257^**^	0.371^**^	0.520^**^	0.405^**^	0.495^**^
4	Impacts and social values				–	0.422^**^	0.149^**^	0.218^**^	0.291^**^	0.351^**^	0.494^**^	0.380^**^	0.462^**^
*Test anxiety*													
5	Test anxiety					-	0.574^**^	0.384^**^	0.493^**^	0.174^**^	0.266^**^	0.172^**^	0.299^**^
*Motivation*													
6	Amotivation						-	0.292^**^	0.437^**^	−0.176^**^	−0.109^*^	−0.188^**^	−0.013
7	External regulation							–	0.392^**^	0.286^**^	0.271^**^	0.241^**^	0.147^**^
8	Introjected regulation								–	0.296^**^	0.259^**^	0.196^**^	0.336^**^
9	Identified regulation									–	0.695^**^	0.700^**^	0.573^**^
10	Knowledge										-	0.773^**^	0.752^**^
11	Accomplishment											–	0.658^**^
12	Stimulation												–

*p≤0.05; **p≤0.01.

Table 4 also presents the correlations between test anxiety and L2 motivation, even though these findings were not initially intended to be investigated by this study. Test anxiety was found to correlate with L2 motivation constructs significantly and positively, with stronger correlations for extrinsic motivation (0.174 ≤ *r* ≤ 0.493, *p* ≤ 0.01), and weaker correlations for intrinsic motivation (0.172 ≤ *r* ≤ 0.299, *p* ≤ 0.01).

#### 5.2.2 Regression analysis

In the next step, multiple regression analyses were further undertaken to test Hypothesis 1 and Hypothesis 2. Students’ institutions were regarded as the covariate. The goodness-of-fit indices for seven final multilevel regression models are reported in Table 5. It is evident from the majority of the evaluated fit indices that most of these models exhibit an adequate fit to the data. Finally, Table 6 presents the estimated parameters derived from these models.

**Table 5:** Fit indices for structural equation modeling (SEM) models.

	Test anxiety	External regulation	Introjected regulation	Identified regulation	Knowledge	Accomplishment	Stimulation
*χ^2^*	4462.655	464.616	481.239	534.381	508.703	512.505	510.334
*df*	1,070	220	220	242	242	242	242
CFI	0.754	0.957	0.956	0.955	0.958	0.958	0.959
TLI	0.741	0.951	0.950	0.948	0.952	0.952	0.953
RMSEA	0.087	0.052	0.053	0.054	0.052	0.052	0.052
SRMR	0.137	0.036	0.036	0.038	0.038	0.038	0.036

Model fit criteria: CFI ⩾ 0.90, TLI ⩾ 0.90, RMSEA ⩽ 0.08.

**Table 6:** Results of multilevel modeling predicting relationships between LAL, test anxiety, and motivation.

	Test anxiety	External regulation	Introjected regulation	Identified regulation	Knowledge	Accomplishment	Stimulation
	*B(SE)*	*B(SE)*	*B(SE)*	*B(SE)*	*B(SE)*	*B(SE)*	*B(SE)*
Institution type	−0.036 (0.04)	0.106 (0.06)	0.009 (0.06)	0.249 (0.07)^***^	0.197 (0.06)**	0.198 (0.07)**	0.178 (0.06)**
Uses of assessments to learn	−0.249 (0.22)	0.040 (0.18)	−0.072 (0.14)	0.460 (0.10)^***^	0.283 (0.12)*	0.398 (0.22)	0.292 (0.08)***
Scores and decision making	0.365 (0.45)	−0.443 (0.27)	−0.008 (0.22)	−0.539 (0.20)**	−0.404 (0.24)	−0.434(0.41)	−0.160 (0.18)
Theoretical knowledge	0.189 (0.15)	0.736 (0.17)^***^	0.018 (0.17)	0.518 (0.18)**	0.612 (0.13)***	0.502(0.21)*	0.491 (0.09)***
Impact and social value	0.040 (0.25)	−0.066 (0.15)	0.363 (0.13)**	0.040 (0.15)	0.126 (0.1)	0.033(0.15)	−0.034 (0.13)

***p ≤ 0.001; **p ≤ 0.01; *p ≤ 0.05.

##### 5.2.2.1 Test anxiety

It was found that after controlling the institution, the latent dimensions of LAL did not significantly predict test anxiety. To be more specific, test anxiety was not significantly predicted by the uses of assessment to learn (*β* = −0.249, Estimate/SE = 0.22, *p* > 0.05), scores and decision making (*β* = 0.365, Estimate/SE = 0.45, *p* > 0.05), theoretical knowledge (*β* = 0.189, Estimate/SE = 0.15, *p* > 0.05), and impact and social value (*β* = 0.04, Estimate/SE = 0.25, *p* > 0.05). The results indicate that students’ LAL does not play a role in either exacerbating or mitigating their test anxiety.

##### 5.2.2.2 Extrinsic motivation

The latent LAL dimensions significantly predict extrinsic motivation. To be specific, theoretical knowledge significantly predicts external regulation (*β* = 0.736, Estimate/SE = 0.17, *p* < 0.001). Therefore, the greater the mastery of theoretical knowledge by students, the higher the external regulation they experience during their English learning. Furthermore, impact and social value significantly and positively predict introjected regulation (*β* = 0.363, Estimate/SE = 0.13, *p* < 0.01). Therefore, the better students comprehend impact and social values, the higher the introjected regulation they experience during their English learning. In addition, the use of assessments to learn (*β* = 0.469, Estimate/SE = 0.10, *p* < 0.001) and theoretical knowledge (*β* = 0.518, Estimate/SE = 0.18, *p* < 0.01) significantly predict identified regulation. Therefore, students’ better understanding of the uses of assessments to learn corresponds to a higher level of identified regulation during their English learning, and greater theoretical knowledge mastery is associated with increased identified regulation during English learning. Surprisingly, students’ scores and decision making could significantly negatively predict students’ identified regulation in language learning (*β* = −0.539, Estimate/SE = 0.20, *p* < 0.01). Consequently, a greater understanding of scores and decision-making by students corresponds to a lower level of identified regulation during their English learning.

##### 5.2.2.3 Intrinsic motivation

The latent dimensions of LAL significantly predict intrinsic motivation. Specifically, the uses of assessments to learn (*β* = 0.283, Estimate/SE = 0.12, *p* < 0.01) and theoretical knowledge (*β* = 0.612, Estimate/SE = 0.13, *p* < 0.001) significantly predict motivation (knowledge). As a result, a more profound comprehension of the uses of assessments for learning leads to an increased motivation for exploring new ideas and acquiring knowledge. Additionally, a higher proficiency in theoretical knowledge among students corresponds to an elevated level of desire for knowledge in the context of their English learning.

Furthermore, theoretical knowledge about language and language learning significantly and positively predicts motivation related to accomplishment (*β* = 0.502, Estimate/SE = 0.21, *p* < 0.05). Therefore, the better students comprehend theoretical knowledge, the higher the motivation for striving to excel in a task or reach a particular goal in English learning.

In addition, the use of assessments to learn (*β* = 0.292, Estimate/SE = 0.08, *p* < 0.001) and theoretical knowledge (*β* = 0.491, Estimate/SE = 0.09, *p* < 0.001) significantly predict motivation that arises from the sensations evoked by engaging in English learning tasks. Therefore, a better understanding of the uses of assessments for learning corresponds to a higher level of motivation for stimulation during English learning, and greater mastery of theoretical knowledge is associated with increased motivation for stimulation during English learning.

##### 5.2.2.4 The impact of institution types

In the Mplus analysis, three types of institutions were coded as 0 for private universities (third-tier university), 1 for key public universities (second-tier university), and 2 for other public universities (third-tier university). From the table, it can be observed that, holding other variables constant, the institution type codes were significantly positively correlated with identified regulation, knowledge, accomplishment, and stimulation. In other words, the higher quality of the institution, the stronger the motivation in students for identified regulation, knowledge, accomplishment, and stimulation. For example, keeping other variables constant, a one-unit increase in institution type (from private university to other public university or from other public university to key public university) is positively correlated with the dependent variable “identified regulation,” resulting in an increase of 0.249 in the value of the dependent variable.

However, no significant relationship was found between institution type and test anxiety, external regulation, and introjected regulation. In other words, when controlling other factors, students from different types of institutions did not show significant differences in their levels of test anxiety, external regulation, and introjected regulation.

## 6 Discussion

The findings indicated that the participants reported a mastery of around 60% of the LAL competence items. To be more specific, they demonstrated comparable levels in their proficiency in their uses of assessments to enhance learning, score interpretation and decision-making abilities, theoretical knowledge about language and language learning, and perception of the impact and social values of language assessment. Moreover, descriptive analyses results indicated that the average test anxiety level among students is 78.55 out of a maximum score of 135. Additionally, these participants displayed stronger intrinsic motivation compared to extrinsic motivation. So far, few studies have been undertaken to explore students’ LAL levels, along with their cognition. One exception is the study by Butler et al. (2021), the interview results of which showed the relatively high-level LAL of primary school students in China. Further research could be done in many areas to gain a greater understanding of students’ LAL, and the results could then be contrasted and compared.

Consistent with Hypothesis 1, the findings indicate that students’ theoretical knowledge about language and language learning can significantly and positively predict students’ external regulation to study English. The reason might be that students with a solid understanding of language and language learning may be more conscious of criteria to assess one’s language proficiency. With this understanding, students’ behavior may be prompted by the assessment criteria and may be more inclined to make efforts in language learning activities in order to earn external acknowledgements, such as high scores (Vansteenkiste et al., 2006). The evidence from previous studies also indicated that the understanding of the language learning and the criteria to assess one’s language proficiency was positively associated with students’ motivation (Jonsson and Svingby, 2007; Brookhart and Chen, 2015).

The findings also indicated that students’ understandings of the social values of language assessment could positively predict their introjected regulation. This finding aligns with the self-determination theory, which proposes that individuals tend to partially internalize the external expectation or societal norms, creating a sense of obligation or pressure to meet these external standards – referred to as introjected regulation (Deci and Ryan, 1986; Pelletier et al., 2001; Dörnyei and Ushioda, 2021). For example, if students believe that excelling in language assessments can earn recognition from peers, they internalize this value, motivating themselves to strive for success in language assessments. On the other hands, students may experience pressure and fear negative judgments from peers due to their poor performance in language assessments. To avoid potential guilt or anxiety arising from such judgments, they may motivate themselves to exert extra effort in language learning activities, resulting in introjected regulation. While they may not fully adopt these social values as their own, they still partially internalize these values, reinforcing their own behaviors.

Another finding was that students’ theoretical knowledge about language and language learning could positively predict their L2 intrinsic motivation (i.e., knowledge, accomplishment, and stimulation) and identified regulation, which is the aspect of extrinsic motivation that is most similar to intrinsic motivation. A possible explanation could be that because students have a deeper understanding of language and language learning, enabling them to choose effective strategies enhance their learning. The improved outcomes of language learning further spark their intrinsic motivation to learn. This finding confirms the theoretical underpinnings of the self-determined theory (Ryan and Deci, 2000a,b,c) and also somewhat agrees with the results of Deeley and Bovill (2017), which found that the improvement of students’ assessment literacy could facilitate them gain better learning achievement, thereby enhancing their learning motivation.

It was also discovered that students’ uses of assessments to improve their language learning could also serve as a positive predictor for their intrinsic motivation (i.e., knowledge, accomplishment, and stimulation) in the L2, as well as identified regulation. The reason could be that as students become adept at utilizing assessments for learning purposes—such as understanding their language proficiency, setting objectives, and preparing for language assessments—they experience a stronger sense of accomplishment and joy, ultimately enhancing their L2 intrinsic motivation. This finding aligns with the findings of previous studies, which indicate that using assessment approaches for learning could contribute to students’ intrinsic motivation (Pat-El et al., 2012; Zoghi and Malmeer, 2013; Weng et al., 2023).

The results additionally indicated that students’ comprehension of scores and decision-making significantly and negatively predicts their identified regulation in language learning, contradicting Hypothesis 2. This could be attributed to the notion that a deep understanding of scores might incline students towards external regulation, implying that scores may become a prominent motivating factor for language learning. The emphasis on scores may divert students’ attention from the personal significance they attribute to assessments, thereby adversely affecting identified regulation. This finding is to some extent supported by previous research, which indicate that extrinsic rewards such as scores can undermine individuals’ intrinsic motivation (Lepper et al., 1973,Deci and Ryan, 1985b; Butler and Nisan, 1986; Deci et al., 1999; Ryan and Deci, 2000c; Chamberlin et al., 2023).

Additionally, contrary to Hypothesis 1, the results indicated that the students’ LAL could not significantly predict their test anxiety when taking language tests. The finding contrasts with a prior study, which was conducted with Germany university students and found that anxious signals and behaviors could be induced by the recognition of the significance of language acquisition or a specific test (Bertrams et al., 2010). The discrepancy may arise from the fact that while acknowledging the importance of language learning and assessment could contribute to test anxiety, there are multiple other factors, such as the test environment, difficulty level, test type, language proficiency, etc., that may simultaneously exert stronger influences on test anxiety.

From the above observation, significant correlations emerge between the institutional quality and students’ intrinsic motivation, as well as the identified regulation, which is close to intrinsic motivation. In Chinese context, students typically need to achieve superior results in the Chinese National College Entrance Examination (Gaokao) to gain admission to more prestigious institutions. In other words, students with better academic performance tend to exhibit higher levels of identified regulation and intrinsic motivation (knowledge, accomplishment, and stimulation). This finding is consistent with previous research as well as the empirical evidence (Trevino and DeFreitas, 2014; Froiland and Worrell, 2016; Dörnyei and Ushioda, 2021), which suggests that, in comparison to extrinsic motivation, intrinsic motivation is more effective in eliciting sustained effort from students in their learning endeavors and overcoming challenges, ultimately leading to superior academic outcomes.

## 7 Conclusions, limitations, and future directions

The present study investigates the LAL of 415 university students in China, and the potential influences of students’ LAL on their test anxiety and L2 motivation. In this study, students reported a mastery of around 60% of the LAL competence items. Furthermore, students’ LAL dimensions demonstrated a positive predictive relationship with their motivation in L2 learning. However, their LAL did not have a significant predictive impact on their test anxiety. So far, scant attention has been paid to the effects of students’ LAL on their test anxiety and motivation that are relevant to language learning. This study contributes to this significant yet generally neglected area and provides evidence for future development in students’ LAL.

This study contributes to the current understanding of LAL. By examining university students’ LAL and revealing their impacts on students’ test anxiety and motivation, this study provides a research-based response to the call made by Smith et al. (2013) to give greater consideration to LAL and demonstrates that LAL can exert an important influence on students’ language learning process. Moreover, this study highlights the potential advantages of considering LAL not only as knowledge and skills of language assessment, but also as a source of language learning motivation. LAL serves as a link between teaching, learning, and assessment. Specifically, the development of LAL could enhance students’ intrinsic motivation, an important factor that sustains their language learning process and improves learning performance (Dörnyei and Ushioda, 2021). Consequently, incorporating LAL into language learning practices can maximize the effectiveness of students’ language learning efforts.

The findings of this study carry significant implications for the teaching and learning of L2. Teachers can introduce students to the culture and theories about language learning and assessment in language courses, fostering a positive perception of these aspects. Additionally, teachers can engage discussions with students regarding the objectives and rationale behind various language assessments, thereby creating a meaningful, transparent, and fair assessment environment. Moreover, teachers should guide students in interpreting their assessment scores, helping them identify their own strengths and weaknesses and adjust their learning plans accordingly. This approach enables students to experience the positive changes of assessments, fostering positive expectations. Furthermore, it is crucial for teachers to enhance their own LAL, as teachers’ LAL serves as a vital source of students’ LAL. Teacher educators should also consider incorporating the assessment literacy components into future teacher training programs. Finally, students could use the research findings as a guide to deliberately improve their own LAL.

This study is not exempt from limitations. First, the data for this study were collected from a sample of Chinese university students, which limit the generalizability of the findings to other populations, such as high school students or students from other contexts. Second, the study only included 415 university students, who may not be representative of the entire population of university students in China. To improve the generalizability of the findings, a larger sample size selected through stratified sampling could be employed. Third, the levels of students’ LAL, test anxiety, and L2 motivation were obtained from students’ self-reported data, which may impede the objectivity. Students may choose the questionnaire responses that are more in line with social desirability, potentially influencing the accuracy of the data. Fourth, this study examined the relationship between students’ LAL and individual factors including test anxiety and motivation through seven separate regression analyses. For future studies, a more comprehensive Structural Equation Modeling model could be employed to illustrate the relationship and interaction among these variables.

This research has identified several issues that warrant further investigation. While this study has explored the impact of LAL dimensions on students’ test anxiety and L2 motivation, future studies could expand the focus to LAL’s influences on other cognitive factors in language learning, such as students’ self-efficacy and their attitudes toward L2. Furthermore, this study relied solely on quantitative questionnaire data, which collects a large number of responses in a short period of time but may fail to capture the in-depth perspectives of students. Future studies could incorporate qualitative data, such as interviews and observations, to gain deeper insights from students and other stakeholders. In addition, the emphasis of this research is on university students in China, where the assessment culture is exam oriented. It would be meaningful to carried out studies in different assessment cultures to broaden our understanding.

## Data availability statement

The raw data supporting the conclusions of this article will be made available by the authors, without undue reservation.

## Ethics statement

Ethical approval was not required for the studies involving humans in accordance with the local legislation and institutional requirements. The studies were conducted in accordance with the local legislation and institutional requirements. Written informed consent for participation was not required from the participants or the participants’ legal guardians/next of kin in accordance with the national legislation and institutional requirements.

## Author contributions

FW: Conceptualization, Data curation, Formal analysis, Investigation, Methodology, Project administration, Supervision, Writing – original draft, Writing – review & editing. XL: Data curation, Investigation, Methodology, Formal analysis, Validation, Resources, Writing – review & editing.
